# The effect of maltodextrin edible coating containing pyracantha extract and potassium nano-carbonate on secondary metabolites, antioxidant capacity and microbiological properties of grape during cold storage

**DOI:** 10.1016/j.heliyon.2024.e34123

**Published:** 2024-07-04

**Authors:** Maryam Ebrahimi, Rouhollah Karimi, Amir Daraei Garmakhany

**Affiliations:** a Grape Processing and Preservation Department, Faculty of Agriculture, Research Institute of Grape and Raisin, Malayer University, Malayer, Iran; b Department of Horticulture and Landscape Engineering, Faculty of Agriculture, Malayer University, Malayer, Iran; c Department of Food Science and Technology, Toyserkan Faculty of Engineering and Natural Resources, Bu-Ali Sina University, Hamadan, Iran

**Keywords:** Grape, Edible coating, Pyracantha extract, Potassium nanoparticles, Antioxidant capacity

## Abstract

This study aimed to investigate the postharvest application of edible coating of maltodextrin containing potassium nanoparticles (KNPs; 0–2%) and pyracantha extract (PE; 0–1.5 %) on the maintenance of phenolic compounds, antioxidant capacity and microbial properties of ‘Rishbaba’ grape during 60 days at −1 °C using response surface methodology and central composite design. The results showed that the applied coating on Rishbaba grape maintained total phenol, total flavonoids, total anthocyanin, stilbenes (resveratrol and viniferin) and catechin. That also caused higher antioxidant capacity and lower mold and yeast growth in grape during the storage time. Finally, the best cold storage conditions of ‘Rishbaba’ grape were determined by using the edible maltodextrin coating containing 2 % KNPs and 1.5 % of PE for 34 days with a desirability of 0.728 in terms of investigated Characteristics. The optimized sample has the amount of total phenol (5.79 mg/g), total flavonoid (8.95 mg/g), total anthocyanin (6.48 mg/g) and the greatest ability to inhibit DPPH free radical (42.56 %) and the lowest growth rate of mold and yeast (21 Cfu/g).

## Highlights

• Maltodextrin (MD) containing K NPs and PE maintained higher phenolic compounds.
• Edible coatings preserved resveratrol, viniferin and catechin during storage period.
• Treated grapes retained antioxidant capacity and prevented mold and yeast growth.
• Best storage conditions were obtained with MD + 2 % K NPs +1.5 % of PE for 34 days.

## 1 Introduction

Grape is one of the most widely consumed fruits in the worldwide, and is also known for its health benefits [1]. Fresh grape (*Vitis vinifera* L.) is a non-climacteric fruit that is sensitive to temperature and moisture with short life after harvesting [2]. Is an excellent source of phenolic compounds, tannins and nutrient compounds such as sugars, minerals, vitamins and natural antioxidants [3]. (Resveratrol is one of the dominant compounds of grapes, which can be found in different cultivars with any color, and its anti-inflammatory activity and inhibition of tumorgenesis were observed in mice [4]. ‘Rishbaba’ is a late-ripening seeded table grape with yellow-green colure skin, conical-shaped cluster as well as large, fleshy and slightly firm berries, which is suitable for long-term cold storage [5]. On the other hand, the gray mold created by *Botrytis cinerea* is one of the most important post-harvest diseases that cause many problems for the transportation and storage of grapes over long distances [6]. The infection caused by this mold can be occur even at a low temperature (0.5 °C) and decreased in the quality of the product and its marketability [6]. In order to reduce the postharvest losses of grapes, researchers have used different methods before and after harvest. These include the use of growth regulators [7], controlled atmosphere [8], external γ-aminobutyric acid [1], carbon dioxide [9], ethanol [10], chitosan [11], and sulfur dioxide [12]. Sulfur dioxide is one of the most common methods for disinfection of fresh grapes during storage period. This method has its limitations and problems. The residues of this compound are hazardous to human health, cause symptoms of poisoning and if used in inappropriate concentrations, cause damage to fresh fruits [1]. Therefore, finding a non-toxic, low-cost, and efficient preservation method is crucial to improve the quality and commercial value of grapes.

Edible coatings are thin layers of food that are used on food products and play an important role in their preservation, distribution and marketing [11,13,14]. These coatings are used in various food products, especially in highly perishable products such as horticultural crops, which are used due to some special features such as low price, availability, working characteristics, gas inhibition effect and reduced respiration, resistance to water and microorganisms are considered to improve texture quality, sensory acceptability and also prevent the transfer of flavor-producing compounds [[14], [15], [16]]. Edible coatings are used in liquid form on food products and the food material is immersed in them and formed as layers on the food product. In addition, edible coatings have been introduced as an excellent way to carry additives, which can have an important effect on preserving the constituents of the product and extending its shelf life [14,17]. Maltodextrin are a class of carbohydrates (CHO) produced from a wide range of plant sources. They are produced industrially by enzymatic or acid hydrolysis to starch followed by purification and spray drying. The powders available on the market, mostly white, have high purity and microbiological safety and are used in a wide range of food and beverage products [18]. Most maltodextrins are completely water-soluble and perform other important functions, such as gel formation or cryopreservation control, and are typically used for microencapsulation of food compounds [18,19]. Plants are the most important source of natural antioxidants. Pyracantha is a species of evergreen and thorny shrub from the *Rosaceae* family، with the names firethorn or pyracantha. *Coccinea* is a European species of firethorn that has been cultivated in gardens since the late 16th century [20]. Its fruits are orange to red and glowing. These fruits are used in traditional medicine due to their diuretic properties and strengthening of the immune system and heart [21]. Phytochemical compounds, antioxidant activity and enzyme inhibition of pyracantha extract (PE) were investigated by Sarikurkcu & Tepe [20] and these researchers showed that the ethanolic extract of this plant is rich in phenolics, flavonoids, tannins and saponins. This extract has the ability to inhibit DPPH free radicals and high iron ion regeneration ability, and also has good inhibitory effects on acetylcholine-esterase, butyrylcholinesterase, α-amylase, α-glucosidase and tyrosinase enzymes [20].

Food additives are widely used as preservatives to control pH, taste or quality properties of foods [20]. Among them, various organic and inorganic salts have antimicrobial effect and may be a good alternative to the use of synthetic fungicides. Availability, relatively low cost and high solubility in water are the main benefits of these compounds [15]. Potassium carbonate is a mineral compound (salt) with the formula K_2_CO_3_, which is a white salt that dissolves easily in water and other solvents such as dimethyl sulfoxide. The special properties of this salt can be attributed to high ionic content, good thermal stability, non-volatility, non-flammability and low viscosity, which is also used in environmentally friendly polymers [22]. Also, the potassium ion present in this chemical composition has important effects on chilling tolerance of products and biosynthesis of phenolic compounds and antioxidant capacity of grapes [23].

Nanotechnology has the capacity of producing new materials by minimizing the size of components to a nanometric level. Odetayo et al. [24] investigate the use of edible coatings in the form of nanoparticles such as Zinc oxide nanoparticle (ZnONPs), silver nanoparticles (AgNPs) and Chitosan nanoparticles (CS-NPs) and their effect on the quality and shelf life of fruits. In this review article, the antimicrobial, antifungal and antioxidant properties as well as the potential to extend shelf life of fruits have been discussed [24]. However, there are no studies on the use of KNPs in combination with PE and maltodextrin, especially in grapes.

The main purpose of postharvest technology is qualitative optimization and reduction of losses during the postharvest period. On the other hand, increasing consumer demand for high quality products in terms of health and safety for the environment has led to a significant increase in research on edible coatings [14]. Grapes is one of the important horticultural products that are a good source of phenolic compounds, natural antioxidants and a set of nutrient compounds [3] that during post-harvest storage cause problems in the field of quality loss [1]. The aim of this study was to investigate the effects of maltodextrin edible coating containing PE and KNPs on the preservation of health-giving compounds of ‘Rishbaba’ grape including phenols, flavonoids, anthocyanins and its antioxidant capacity and microbiological properties during the storage time.

## 2 Materials and methods

### 2.1 Preparation materials

*Pyracantha coccinea* fruit was collected from pyracantha trees (a species of evergreen and thorny shrubs from the *Rosaceae* family with orange fruits) on the campus of Malayer University, Iran. Grape clusters of ‘Rishbaba’ cultivar (late-ripening grape variety with large size (7 g) and thick skin berries) were harvested at the commercial maturity stage in the cool hours (5:30 to 7 morning) of the day from the research vineyard of Malayer University, Iran. Healthy and identical clusters were selected in terms of shape, size, color, and clusters with sun burn, shell split were removed. Potassium nanoparticles (KNPs) with food grade (Purity 99.5 and particle size 100 nm) were prepared from Nanotechnology Laboratory of Malayer University, Iran. Maltodextrin powder (soluble in water with DE < 20 and 4 % moisture) and PDA culture medium were also purchased from Merck Company, Germany.

#### 2.1.1 Extraction of PE

For preparation of PE, the fruits were mixed with distilled water (25 g with 100 mL) in ratio of 1:4 at room temperature and after homogenization in ultrasonic device (Ultrasonic homogenizer, 400 W, 20 KHz, topsonics, Lithuania) for 6 min with 400 W power for extraction. After filtering with Watman filter paper No. 1, the resulting extract, after separating the solvent by oven under vacuum at 50 °C, was used in different concentrations to prepare coating solutions [25].

#### 2.1.2 Preparation of coatings and coating operations

Initially, in order to prepare the 10 % maltodextrin solution, after adding its powder to distilled water, stirring was done slowly in laboratory conditions to completely dissolve. In order to dehydrate the maltodextrin biopolymer, stirred for 30 min after dissolution. Finally, by adding three different concentrations of PE (0, 0.75 and 1.5 %w/v) and three different concentrations of KNPs (0, 1 and 2 %w/v), the coating solutions were prepared and again subjected to ultrasonic waves (Ultrasonic homogenizer, 400 W, 20 KHz, topsonics, Lithuania) with a power of 400 W for 5 min. Then, the grape clusters were immersed in each prepared coating solution for 15 min to carry out the coating process. After drying on dryer paper at laboratory temperature, the fruits were placed in polyethylene containers (21 cm × 14 cm x 8 cm) and stored in a refrigerator with −1 ± 0.5 °C and 90 % relative humidity. At certain intervals (0, 30, 60 days), 200 g of the packaged samples were randomly removed from the refrigerator and then evaluated for physiological changes during the storage period [26]. The treatments used in this study are presented in Table 1. After finding the best conditions for grape coating, one group of grape clusters as a control sample (without coating) and one sample under the same optimal conditions in the maximum storage time were examined by Design Expert software.

**Table 1:** Independent variables and their applied levels.

Independent variables		Variables level	
	−1	0	+1
**ST (day)**	0	30	60
**KNPs(%)**	0	1	2
**PE (%)**	0	0.75	1.5

KNPs:Potassium carbonate nanoparticles, PE: pyracantha extract concentration.

### 2.2 Total phenol measurement

The total phenol content was measured by 10%Folin-Ciocalteu (Merck, Germany) reagent. At first, 10 g of fruit tissue (the skin and flesh of the fruit without seed) was applied in 15 mL methanol (Purity 96 %, Merck) to obtain homogeneous solution. After 20 min of centrifugation (Thermo, Japan) at 5056 g, 300 μl of clear supernatant extracts were prepared. Then according to the method of Muzolf-Panek and Waśkiewicz, the total phenolic content was determined by absorption at 765 nm by a spectrophotometer (Varian Cary 300, USA) based on the standard curve of gallic acid [27].

### 2.3 Measurement of stilbenes (resveratrol, viniferin) and flavonoidcatechin

At first, 3 g of each sample was boiled in HCL (0.1 N, Merck) for 25–30 min. Then, they were separated and purified with the help of ethyl acetate and water. The insoluble fraction was dissolved in 80 % methanol and filtered. After filtration, it was injected to a HPLC pump (Crystal 200 series, Unicam, UK) equipped with a Hypersil ODS column (5 μm, 250 mm × 4.6 mm i.d) at 25 °C and a UV–Vis detector, regulated at 254 nm. The mobile phase consisted of dihydrogen potassium phosphate and acetonitrile (80:20 v/v) and flow rate was 1 mL/min. Standards (resveratrol, viniferin, catechin) were purchased from E. Merck and prepared at a concentration of 1 g per 100 mL of pure methanol [28].

### 2.4 Total flavonoid measurement

To measure the amount of total flavonoids, aluminum chloride colorimetric method was used in accordance with the method used by Zhishen et al. with a few changes. After preparing the samples, adsorption at 415 nm was determined by spectrophotometer. The standard quercetin curve was used to calculate this parameter, which was expressed in mg/kg [29].

### 2.5 Total anthocyanin measurement

In order to measure the amount of anthocyanin, first, 0.4 mL of grape extract (extract obtained from grapes by pressing method) was poured into two separate tubes. Then, 3.6 mL of potassium chloride (0.025 M, pH = 1, Sigma-Aldrich) was added to one tube and 3.6 mL of sodium acetate (0.4 M, pH = 5.4, Sigma-Aldrich) was added to the other tube. After mixing, the absorbance rate of the samples was calculated according to equation (1), and at wavelengths of 700 and 510 nm based on the blank sample (distilled water).

(1) $$A={\left(A_{510}-\;A_{700}\right)}_{pH=1}-{\left(A_{510}-\;A_{700}\right)}_{pH=4.5}$$

Total anthocyanin (TAC) was calculated according to equation (2) and expressed in terms of cyanidin-3-glucoside (mg/100 g) [30].

(2) $$TAC=\frac{\left(A\times MW\times DF\times 100\right)}{MA}$$

in equation 2, A: absorption rate calculated from equation (1), MW: molecular weigh (499.2), DF: dilution factor (10), MA: molar absorptivity (26900).

### 2.6 Determination of DPPH scavenging activity

In this method, the reduction power of grape extract on the free radical DPPH and its color change from purple to yellow at 517 nm wavelength were investigated. In this way, 5 mL of grape extract was mixed with 1 mL methanolic DPPH solution (1 mM, Merck) and then stored for 30 min at ambient temperature and dark conditions and finally the absorption rate of samples was read at 517 nm [31].

### 2.7 Microbial analysis

At first, 10 g of each sample was mixed with 90 mL sterile physiology serum and homogenized, after preparation of different dilutes, 1 mL of each dilution was transferred to Petri dish containing PDA medium and cultured by surface method and incubated at 25 °C for 5 days. Samples were prepared in three replications and only 30–300 colony forming units (cfu/g) were considered [32].

### 2.8 Statistical analysis

Data analysis of secondary metabolites, antioxidant capacity and microbiological properties of ‘Rishbaba’ grape under the influence of PE, concentration of KNPs and storage time using response surface methodology in the form of Central Composite Design (CCD) was performed using Design Expert software (6.0.2). For this purpose, a central composite design with 3 levels and 5 replications at the center point was used to investigate the desired properties. In this research, the independent variables, storage time (_X1_), concentration of KNPs (_X2_) and concentration of PE (_X3_) were deduced from the initial tests. To evaluate the behavior of the response levels, a quadratic polynomial equation was fitted for each independent variable (Equation (3))

(3) $$Y=\beta_{0}+\sum_{i=1}^{3}\beta_{i}X_{i}+\sum_{i=1}^{3}\beta_{ii}X_{ii}^{2}+\sum_{i=1}^{2}\sum_{j=i+1}^{3}\beta_{ij}X_{i}X_{j}$$

where y is the estimated response (amount of total phenol, resveratrol, viniferin, catechin, total flavonoid, total anthocyanin, the ability to inhibit DPPH free radicals, mold and yeast growth), b0, bi, bii and bij are constant coefficients and xi, xj express The independent variables are coded. The quality and accuracy of the regression model and the appropriateness of the fit are determined by the parameters of the model analysis, the fit weakness and the coefficient of determination. The analysis of variance of the test data is shown in Table 3.

## 3 Results and discussion

### 3.1 Total phenolic content

**Fig. 1:**
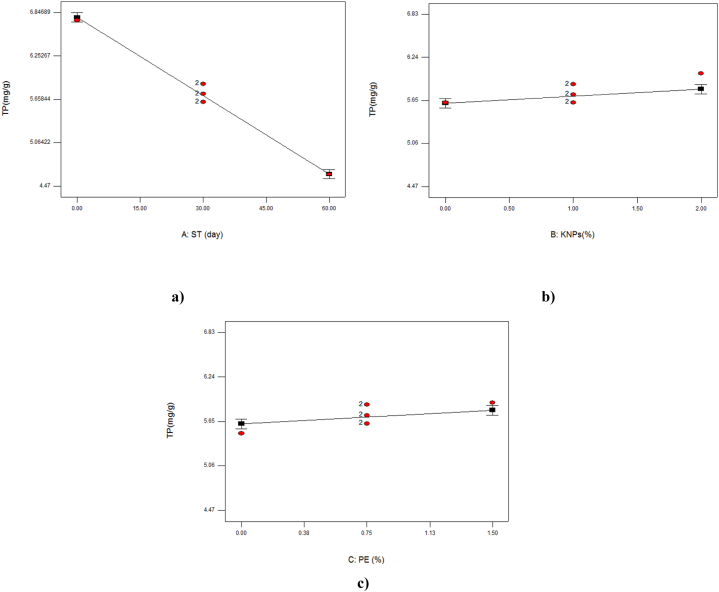
The effect of storage time (ST; a) percentage of potassium nanoparticles (KNPs; b) and percentage of pyracantha extract (PE; c) on the total phenol (TP) content of Rish- Baba grapes during cold storage.

According to the analysis table of variance (Table 3), only the linear parameters of the studied variables *i.e.* storage time, percentage of KNPs and PE concentration were significant on the total phenol content of samples. As in Fig. 1a and Table 2 can be seen the amount of total phenol of grape decreased with the increase of storage time, but preserved with the increase of KNPs and PE (Fig. 1 a and b). The highest amount of phenolic compounds (6.83 mg/g) was observed in the sample coated with 2 % KNPs and 1.5 % PE. The increase of polyphenols can be attributed to the stimulation of phenylalanine ammonia lyase activity, which plays an important role in the production of polyphenolic compounds [33]. After harvesting fruits, the total phenolic concentration remains either constant or decreases slowly, and the effect of unique phenolic compounds on browning rates varies [34]. Edible coatings have been proven to create a barrier on the surface of products, resulting in reduced oxygen and thus reduced oxidation of phenols [35]. In Table 4, some of the researchers' findings are given that were consistent with the results of this study.

**Table 2:** Experimental designed experimental data.

Run	Variables			Responses							
	ST (day)	KNPs(%)	PE (%)	TP (mg/g)	Resveratrol (mg/g)	Viniferin (mg/g)	catechin (mg/g)	TF (mg/g)	Anthocyanin (mg/g)	DPPH FRSA(%)	Microbial Test(cfu/g)
1	30	2	0.75	6.02	6.80	8.57	6.26	8.94	6.61	40.50	26
2	30	1	0.75	5.87	10.82	7.64	5.85	8.68	6.34	40.19	63
3	0	0	0	6.65	11.67	9.15	6.75	9.14	7.30	44.17	105
4	30	1	0.75	5.73	10.68	7.73	5.92	8.83	6.15	40.24	65
5	0	1	0.75	6.74	11.79	9.22	6.89	9.18	7.35	44.26	56
6	0	2	1.5	6.83	11.86	9.30	6.98	9.25	7.39	44.32	27
7	0	0	1.5	6.70	11.73	9.11	6.85	9.08	7.17	44.22	55
8	60	1	0.75	4.63	9.40	6.11	4.79	7.19	4.46	35.02	166
9	30	1	0.75	5.62	10.90	8.00	5.90	8.86	6.41	39.98	64
10	30	1	1.5	5.90	11.08	8.23	6.01	8.89	6.47	40.38	40
11	60	0	0	4.47	8.72	5.75	4.57	6.99	4.14	34.50	247
12	30	1	0.75	5.87	10.87	7.86	5.82	8.68	6.34	40.19	60
13	30	1	0	5.49	10.24	7.55	5.58	8.50	5.96	39.62	72
14	30	1	0.75	5.73	10.92	7.73	5.78	8.83	6.15	40.24	62
15	60	2	0	4.50	9.71	6.30	5.11	7.24	4.62	35.30	119
16	30	1	0.75	5.62	10.75	7.50	5.83	8.86	6.41	39.98	61
17	60	2	1.5	4.82	9.95	6.59	5.26	7.63	4.90	35.78	104
18	60	0	1.5	4.56	9.09	5.97	4.71	7.07	4.29	34.84	178
19	30	0	0.75	5.62	10.51	7.80	5.77	8.63	6.12	39.90	105
20	0	2	0	6.80	11.84	9.26	6.96	9.10	7.24	44.14	21

ST: Storage Time, KNPs: Potassium carbonate nanoparticles, PE: pyracantha extract concentration, TP: Total phenol, TF: Total Flavonoid, DPPH FRSP: DPPH Free Radical Scavenging Activity.

**Table 3:** Analysis of variance of the second order for all responses.

Source	Responses							
	TP (mg/g)	Resveratrol (mg/g)	Viniferin (mg/g)	Catechin (mg/g)	TF (mg/g)	Anthocyanin (mg/g)	DPPH (%)	Microbial test (Cfu/g)
Model	11.71^b^	16.28^b^	24.11^b^	10.55^b^	11.34^b^	21.12^b^	211.02^b^	61682.9^b^
X_1_	11.53^b^	14.45^b^	23.47^b^	9.98^b^	9.27^b^	19.71^b^	208.57^b^	30250.0^b^
X_2_	0.094^a^	0.90^b^	0.50^b^	0.37^b^	0.16^b^	0.30^b^	0.58^b^	15444.9^b^
X_3_	0.081^a^	0.23^a^	0.14^ns^	0.071^a^	0.090^b^	0.092^a^	0.33^b^	2560.0^b^
X_1_^2^	–	0.13^a^	–	0.002^ns^	0.89^b^	0.43^b^	0.59^b^	6420.3^b^
X_2_^2^	–	0.041^ns^	–	0.059^a^	0.0028^ns^	0.011^ns^	0.026^ns^	21.8^b^
X_3_^2^	–	0.065^ns^	–	0.015^ns^	0.0091^ns^	0.021^ns^	0.029^ns^	122.8^b^
X_1_ X_2_	–	0.30^b^	–	0.070^a^	0.058^a^	0.11^a^	0.35^b^	1012.5^b^
X_1_ X_3_	–	0.035^ns^	–	0.003^ns^	0.018^ns^	0.021^ns^	0.044^ns^	200.0^b^
X_2_X_3_	–	0.0036^ns^	–	0.0006^ns^	0.034^ns^	0.021^ns^	0.009^ns^	1512.5^b^
Residual	0.20	0.26	0.93	0.072	0.073	0.16	0.19	18.2
Lack of fit	0.13^ns^	0.22^ns^	0.78^ns^	0.059^ns^	0.036^ns^	0.083^ns^	0.12^ns^	0.7^ns^
Pure Error	0.063	0.043	0.15	0.014	0.037	0.072	0.076	17.5
Core Total	11.91	16.54	25.05	10.63	11.41	21.28	211.21	61701.2

TP: Total Phenol (mg/g), TF: Total Flavonoid(mg/g), Significance level.

^ns^Nonsignificant.

a p < 0.05.

b p < 0.01.

**Table 4:** Studies according to the results of this research.

Features	References	Test conditions	Results
Total phenol, resveratrol, viniferin, catechin, total flavonoid, anthocyanins	Eshghi et al., 2022	Using chitosan and gum arabic for coating of the grapes	Reduction of total phenol, resveratrol and viniferin by increasing storage time and maintaining more of these compounds by using coating
	Yang et al., 2022	Application of turmeric and green tea extract in strawberry edible coating	More preservation of phenolic compounds in strawberries during the storage time
	Awad et al., 2015	Postharvest *trans*-resveratrol and glycine betaine treatments affect quality and antioxidant of ‘El-Bayadi'table grapes after storage and shelf life.	More preservation of antioxidant compounds in grapes over time
	Flamini et al., 2016	–	The decrease of viniferin during storage time due to the growth of mold and yeast in grapes
	Rocha-Parra et al., 2016	Using gum arabic with maltodextrin to coat red wine	Reduction of catechin content over time and its preservation by coating
	Albuquerque et al., 2017	–	The reason for the decrease of catechin over time was attributed to the decrease in pH
	Xing et al., 2021	Effect of chitosan/nano-TiO2 composite coating on the postharvest quality of blueberry fruit.	Preservation of flavonoids in blueberry over time using edible coatings
	Muche et al., 2018	preservation of grape juice of different varieties during storage	Reduction of anthocyanins with time during storage
DPPH	De Bruno et al., 2023	Application of Edible Coating Enriched with Natural Antioxidant Extract & Bergamot Essential Oil on the Shelf Life of Strawberries.	The decrease of DPPH with the passage of time that the use of the coating slowed down this process
Mold and yeast growth	Xu et al., 2007	Using edible coatings such as chitosan in grape preservation	Reducing mold and yeast growth using edible coatings
	Khan et al., 2019	The use of edible coatings containing antimicrobial compounds in strawberry fruit	Preventing the growth and activity of microorganisms

### 3.2 Resveratrol content

**Fig. 2:**
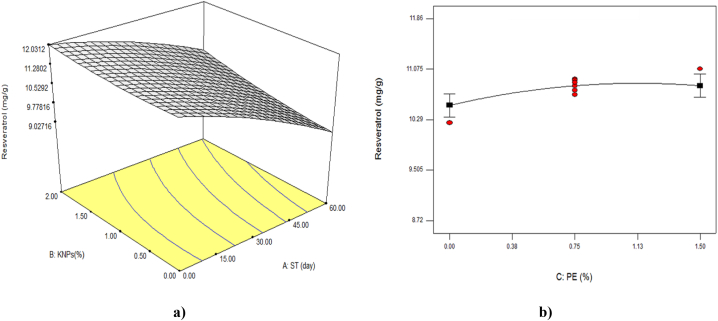
The effect of storage time (ST) and percentage of potassium nanoparticles (KNPs; a) and percentage of pyracantha extract (PE; b) on the resveratrol content of Rish -Baba grapes during cold storage.

Table 3 showed that all linear parameters had significant effect on resveratrol content of grapes during storage time (P < 0.01). While among the quadratic parameters, only storage time (P < 0.05) and between interactions, interaction of storage time and concentration of KNPs (P < 0.01) were significant on this property. The results of Fig. a2 and Table 2 showed that the amount of resveratrol decreased with increasing the storage time of grapes and decreasing the concentration of KNPs in the coating formulation. In other words, in higher concentrations of KNPs with increasing storage time, the reduction of resveratrol was less. With increasing the concentration of PE, the resveratrol content of samples increased slightly (Fig. 2b). The highest amount of resveratrol (9.95 mg/g) on the last day of storage was related to the sample coated with 2 % KNPs and 1.5 % PE. Studies have shown that the use of edible coatings leads to the preservation of phenolic compounds during storage time. For example coating strawberry fruit with Aloe Vera gel enriched with ascorbic acid, calcium lactate and cinnamon essential oil protected the phenolic compounds [36]. At least, some of the improved content of resveratrol in the present study can be related to the role of potassium in the biosynthesis of phenolic compounds [37], as well as the antioxidant effect of PE [38], which has led to the maintenance of this stilbene content in the samples treated with these compounds. Table 5, which shows the equation related to the quadratic model on the amount of resveratrol, indicated that the linear parameter of storage time had a greater effect on this characteristic.

**Table 5:** Designed equation models for the selected dependent variables.

Number	Dependent variable	Equation	R^2^	R^2^-adj	CV
1	Total Phenol	Y = +5.71–1.07 X_1_+0.097 X_2_ +0.090 X_3_	0.98	0.98	1.94
2	Resveratrol	Y = +10.82–1.20 X_1_+0.30 X_2_+0.15 X_3_-0.22 X_1_^2^+0.12 X_2_^2^-0.15 X_3_^2^+0.19 X_1_X_2_+0.066 X_1_ X_3_-0.021 X_2_ X_3_	0.98	0.97	1.51
3	Viniferin	Y = +7.77–1.53 X_1_+0.22 X_2_+0.12 X_3_	0.96	0.96	3.11
4	Catechin	Y = +5.86–1.00 X_1_+0.19 X_2_+0.084 X_3_-0.029 X_1_^2^+0.15 X_2_^2^-0.074 X_3_^2^+0.094 X_1_ X_2_+0.021 X_1_ X_3 –_ 0.008 X_2_ X_3_	0.99	0.99	1.45
5	Total Flavonoid	Y = +8.78–0.96 X_1_+0.13 X_2_+0.095 X_3_-0.57 X_1_^2^+0.032 X_2_^2^-0.058 X_3_^2^+0.085 X_1_ X_2_+0.047 X_1_ X_3_+0.065 X_2_ X_3_	0.99	0.99	1.01
6	Anthocyanin	Y = +6.30–1.40 X_1_ +0.17 X_2_+0.096 X_3_ -0.40 X_1_^2^+0.064 X_2_^2^-0.086 X_3_^3^+0.12 X_1_ X_2_+0.051 X_1_X_3_+0.051X_2_X_3_	0.99	0.97	2.05
7	DPPH Free Radical Scavenging Activity	Y = +40.12–4.57 X_1_+0.24 X_2_+0.18 X_3_-0.46 X_1_^2^+0.097 X_2_^2^-0.1 X_3_^3^+0.21 X_1_ X_2_+0.074 X_1_ X_3_ + 0.034 X_2_ X_3_	0.99	0.99	0.35
8	Microbial test	Y = +62.57 + 55.X_1_-39.3 X_2_-16 X_3_+48.32 X_1_^2^+2.82 X_2_^2^-6.68 X_3_^2^-11.25 X_1_ X_2_ – 5 X_1_X_3_+13.75 X_2_ X_3_	0.99	0.99	1.59

### 3.3 Viniferin content

**Fig. 3:**
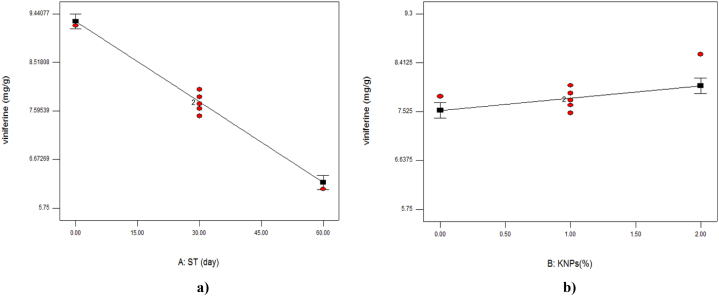
The effect of storage time (ST; a) and percentage of potassium nanoparticles (KNPs; b) on the viniferin content of Rish- Baba grapes during cold storage.

The results in Table 3 showed that only linear parameters of storage time and concentration of KNPs on the amount of viniferin were significant (P < 0.01). In the investigation of the effect of coatings on viniferin content in ‘Rishbaba’ grape was determined that with increasing the cold storage time, the amount of viniferin decreased (Table 2, Fig. 3). As Fig. 3b showed with increasing the percentage of KNPs, the amount of viniferin preserved in the fruit tissue increased. Like resveratrol, the maximum amount of viniferin on the last day of storage was related to the sample coated with 2 % KNPs and 1.5 % PE, which was probably due to preventing the growth of microorganisms and less degradation of this compound. On the other hand, equation (3) in Table 5 showed that the linear parameter of storage time had the greatest effect on the amount of viniferin. In Table 4, some of the researchers' studies that corresponded with the results of this study are given.

### 3.4 Catechin content

**Fig. 4:**
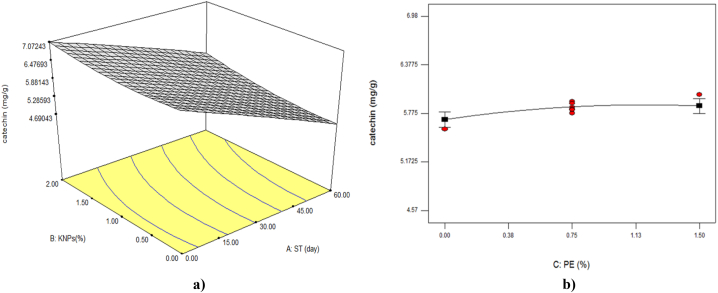
The effect of storage time (ST) and percentage of potassium nanoparticles (KNPs; a) and percentage of pyracantha extract (PE; b) on the catechin content of Rish-Baba grapes during cold storage.

The proposed model was significant (P < 0.01) in the study of catechin changes (Table 3). On the other hand, it was found that among the interaction parameters only the interaction between storage time and the concentration of KNPs on the content of this compound was significant (P < 0.05). As shown in Table 2 and Fig. 4, it was found that the amount of catechin in the fruit decreased with the increase of cold storage time and decreased of KNPs. These changes were due to the protective effect of the edible coating of maltodextrin containing KNPs and PE, which was caused more catechin preservation in the coated fruit tissue compared to the control sample. Also, the findings indicated that the greatest effect on the amount of catechin was related to the linear parameter of storage time (Table 5).

### 3.5 Total flavonoid content

**Fig. 5:**
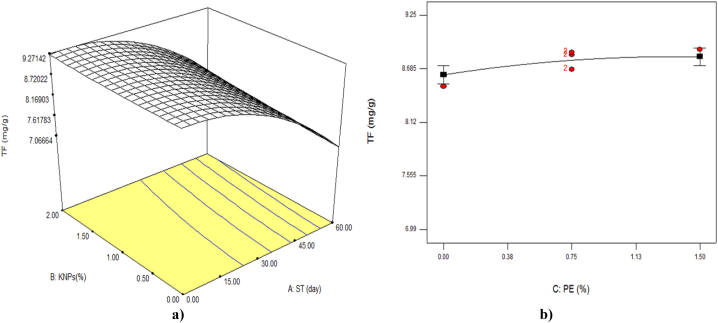
The effect of storage time (ST)and percentage of potassium nanoparticles (KNPs; a) and percentage of pyracantha extract (PE; b) on the total flavonoid (TF) content of Rish-Baba grapes during cold storage.

The results in Table 3 showed that all linear parameters and quadratic parameter of storage time (x_1_^2^) were significant on the amount of flavonoids (P < 0.01). Between the interaction effects only the interaction storage time with the concentration of KNPs had a significant effect on this feature (P < 0.05). Fig. 5a and Table 2 showed that in low concentrations of KNPs and with increasing of storage time the amount of flavonoids decreased. In other words, KNPs had protective effect on flavonoids. The findings of this study also showed that PE increased the retention of total flavonoids in fruit tissue during cold storage and increased its percentage from 0 to 1.5 % had a greater effect on the preservation of flavonoids (Fig. 5b). After 60 days of storage, the lowest amount of total flavonoids (6.99 mg/g) belonged to the sample without KNPS and PE. The equation given in Table 4 showed the greater effect of the linear parameter of storage time on the total flavonoid of samples. The application of the edible coating of maltodextrin containing PE and KNPs has prevented the production of abiotic stresses and by protecting flavonoids leads to slower degradation of these secondary metabolites, which play an important role in inhibiting free radicals.

### 3.6 Total anthocyanin content

**Fig. 6:**
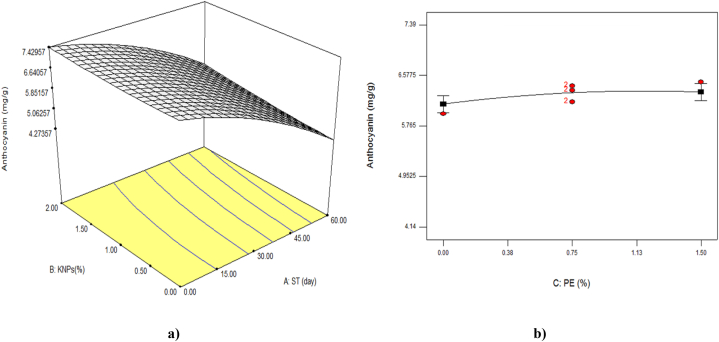
The effect of storage time (ST)and percentage of potassium nanoparticles (KNPs; a) and percentage of pyracantha extract (PE; b) on the total anthocyanin content of Rish-Baba grapes during cold storage.

Anthocyanins are polyphenolic compounds that give a full range of colors from red to black to many fruits, flowers and vegetables. These pigments not only have aesthetic appeal, but they also have other physiological roles in plants such as protecting them against pathogens and abiotic stress conditions [39]. Table 3 showed that all linear parameters studied were significant on total anthocyanin (P < 0.01). However, only the quadratic parameter of storage time (P < 0.01), and among the interaction parameters, the interaction effect of storage time with the concentration of KNPs on the amount of total anthocyanin was significant (P < 0.05). As shown in Fig. 6, during the cold storage time, the amount of total anthocyanin decreased with increasing storage time and decreasing KNPs and PE (P < 0.01). So that on the 60th day of storage, the lowest amount of total anthocyanin (4.14 mg/g) was observed in the sample without KNPs and PE. During the storage time, the enzymes activities cause oxidation and destruction of anthocyanins. The difference of coated treatments in the maintenance of anthocyanins can be attributed to their ability to delay aging and decreased activity of anthocyanins degrading enzymes. In this study, the trend of reducing anthocyanins in coated samples was milder than the control sample, which was consistent with the findings of other researchers [40]. Low-temperature processing and storage can improve the stability of anthocyanins, but light is usually detrimental to the stability of this compound. On the other hand, it was found that like all the measured characteristics, the linear parameter of storage time had the greatest effect on the total anthocyanin content (Table 5; Fig. 6).

### 3.7 Antioxidant capacity by DPPH method

**Fig. 7:**
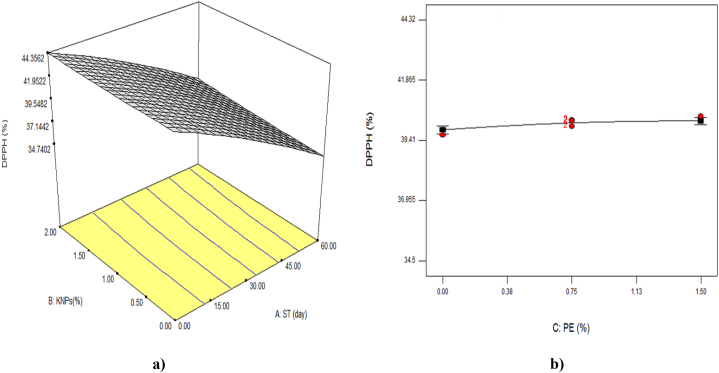
The effect of storage time (ST) and percentage of potassium nanoparticles (KNPs; a) and percentage of pyracantha extract (PE; b) on the DPPH of Rish-Baba grapes during cold storage.

Table 3 showed that the interaction between storage time and concentration of KNPs on the antioxidant capacity of samples by DPPH method was significant (P < 0.01). As shown in Fig. 7, the ability to inhibit DPPH free radicals decreased with increasing storage time and decreasing PE in the used coating. According to equation 7 of Table 5, the greatest impact on this feature was related to the linear parameter of the storage time. In general, the reduction of anthocyanins, phenols and flavonoids is one of the reasons for the decrease in antioxidant activity, because these compounds have antioxidant properties that will oxidize over time and the antioxidant activity will also decrease. Another reason for the reduction of this factor is its role - protecting cells from damage caused by free radicals [41]. As a result of increased oxidative metabolism, ROS increase, which can destroy biological membranes. In order to prevent cell damage caused by ROS such as superoxide anion and H_2_O_2_, plants use various antioxidant enzymes such as superoxidase dismutase, catalase, ascorbate peroxidase, glutathione reductase, and also use non-enzymatic antioxidants with low molecular weight such as ascorbic acid, glutathione, vitamin E, carotenoids and flavonoids [1,42]. Severe reduction in postharvest antioxidant compounds may be due to postharvest stress and low storage temperatures [5]. Treatments that reduce respiration and ethylene production and thus decrease the rate of aging, including the application of edible coatings containing different compounds that slow the production of free radicals and thus reduce the consumption of antioxidants [16].

**Fig. 8:**
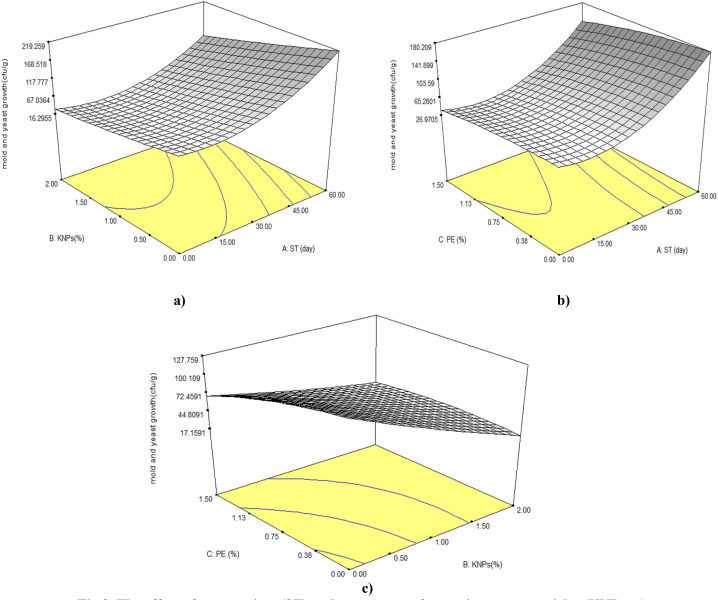
The effect of storage time (ST) and percentage of potassium nanoparticles (KNPs; a), storage time and percentage of pyracantha extract (PE; b), KNPs and PE (c) on mold and yeast growth on the Rish-Baba grapes during cold storage.

### 3.8 Growth of mold and yeast

Analysis of variance of the data in Table 3 showed that all linear, quadratic and interaction parameters had a significant effect on the growth of mold and yeast (P < 0.01). The results showed that with increasing the time of cold storage and approaching the end of the days, the activity of mold and yeast increased significantly and the number of counted colonies was higher. Whereas from the beginning to the 30th day, there was no significant difference in the growth of mold and yeast in the control sample with the coated samples (Fig. 8 and Table 2). In the present study, the results showed that application of coatings containing KNPs and PE reduced the growth of mold and yeast on grapes during the storage time and with increasing their percentage, this inhibitory effect increased. In other words, the growth of mold and yeast was less in the less time of storage and the application of KNPs and PE in the coating. The simultaneous application of KNPs and PE in the coating had an intensifying effect of inhibiting the growth of mold and yeast. The mechanism of inhibition of microorganism growth by edible coating is that the coatings used have reduced respiration and fruit permeability. The results indicate that the PE has antimicrobial activity and is also antioxidant, preventing the destruction of phenolic compounds such as resveratrol, which itself has antimicrobial properties and delays the growth of mold and yeast [11]. On the other hand, equation 8 mentioned in Table 5 showed that, like all the studied features, the linear parameter of storage time had the greatest effect on the number of mold and yeast in the samples.

### 3.9 Optimization the storage conditions of coated grapes

In order to find the best storage conditions for grapes coated with maltodextrin containing KNPs and PE, the storage time in the range of 0–60 days, the concentration of KNPs at the level of 0–2 %, and the concentration of PE at the level of 0–1.5 % were determined. The storage process of ‘Rishbaba’ grapes under mentioned conditions was optimized in order to achieve the maximum amount of secondary metabolites and antioxidant capacity and the lowest amount of mold and yeast growth. According to the desired conditions, the proposed solutions were presented with the highest desirability and the utility of 0.728 were determined as the most suitable case to achieve the optimal conditions. Table 6 shows the data obtained from the optimal conditions, the control sample (without coating) and also the experimental data obtained from the optimal conditions. The results showed that with the application of maltodextrin coating containing 2 % KNPs and 1.5 % PE and 34 days storage time, the product had the best quality in terms of the amount of phenolic compounds, flavonoids, phenolic acids, catechin and total anthocyanin. And the lowest amount of mold and yeast growth was obtained. It was found that except for the number of mold and yeast, the amount of other characteristics in the control sample was lower than the optimal sample. Also, the presented models predicted the results of the measured characteristics with acceptable accuracy.

**Table 6:** Optimized condition, experimental data and control sample achieved in this study.

Variables		ST (day)	KNPs (%)	PE (%)	TP (mg/g)	TF (mg/g)	Anthocyanin (mg/g)	DPPH (%)	Microbial test (Cfu/g)
Goal		0–60	0–2	0–1.5	maximize	maximize	maximize	maximize	minimize
Optimization	34		2	1.5	5.79	8.95	6.48	42.56	21.00
Experimental data	34		2	1.5	5.00	8.05	6.00	39.00	39.00
Control	34		2	1.5	3.21	6.25	4.07	31.39	280.00

All measured responses were optimized with a desirability of 0.728. ST: Storage Time, KNPs: Potassium nanoparticles, PE: pyracantha extract concentration, TP: Total phenol, TF: Total Flavonoid, DPPH FRSA: DPPH Free Radical Scavenging Activity.

### 3.10 Investigating the correlation of the measured characteristics

Table 7 shows that all the measured characteristics have a positive correlation with each other except the number of mold and yeast, which indicates that all the studied compounds, prevented the growth of mold and yeast. On the other hand, it was found that among the characteristics measured, the highest correlation was between the two characteristics of total phenol and the ability to inhibit DPPH free radicals, and this correlation was positive. The lowest correlation was between the ability to inhibit DPPH free radicals with the number of mold and yeast, which was negative.

**Table 7:** Correlation between measured characteristics.

	Total phenol	Resveratrol	Viniferin	catechin	Total flavonoids	total anthocyanins	DPPH	mold and yeast
Total phenol	–	+0.938	+0.972	+0.962	+0.859	+0.958	+0.986	−0.591
Resveratrol	+0.938	–	+0.964	+0.960	+0.899	+0.963	+0.930	−0.755
Vinifrine	+0.972	+0.964	–	+0.977	+0.879	+0.966	+0.967	−0.648
catechin	+0.962	+0.960	+0.960	–	+0.828	+0.942	+0.962	−0.631
Total flavonoids	+0.859	+0.899	+0.879	+0.828	–	+0.952	+0.880	−0.755
total anthocyanins	+0.958	+0.963	+0.966	+0.942	+0.952	–	+0.960	−0.696
DPPH	+0.986	+0.930	+0.967	+0.962	+0.880	+0.960	–	−0.584
mold and yeast	−0.591	−0.755	−0.648	−0.631	−0.755	−0.696	−0.584	–

The data includes the degree of correlation and the + and - signs respectively indicate the relationship between the features in terms of positive and negative correlation.

## 4 Conclusion

Coating of ‘Rishbaba’ grapes with edible coating of maltodextrin containing KNPs and PE as a post-harvest storage method caused preservation of secondary metabolites, antioxidant capacity, and reduction of mold and yeast growth compared to the control sample during cold storage time. In this study, the reduction of total phenol, phenolic acids (resveratrol, viniferin), flavonoids, anthocyanins and antioxidant capacity in the coated samples was milder than the control sample. So that the highest amount of total phenol (4.82 mg/g), total flavonoid (7.63 mg/g), total anthocyanin (4.90 mg/g) on the last day of storage corresponds to the sample coated with a coating containing 2 % KNPs and 1.5 % PE reported. On the other hand, the results showed that PE has antimicrobial activity and also has antioxidant properties and prevents degradation of phenolic compounds such as resveratrol, which has antimicrobial properties and delays the growth of mold and yeast. Therefore, the application of maltodextrin coating containing 2 % KNPs and 1.5 % of PE can be suggested as an appropriate strategy for reducing postharvest damage by microorganisms and also preserving compounds with potentially beneficial effects on human health.

## Data availability statement

The datasets generated and analyzed during this study can be availed upon request from the corresponding author.

## CRediT authorship contribution statement

**Maryam Ebrahimi:** Writing – review & editing, Writing – original draft, Methodology, Investigation, Formal analysis, Data curation, Conceptualization. **Rouhollah Karimi:** Writing – review & editing, Writing – original draft, Resources, Project administration, Funding acquisition, Conceptualization. **Amir Daraei Garmakhany:** Writing – review & editing, Writing – original draft, Software, Methodology, Investigation, Formal analysis, Data curation.

## Declaration of competing interest

The authors declare that there are no conﬂicts of interest.
